# The Occupational Challenges and Responses of International Graduate Students in South Korea: A Scoping Review

**DOI:** 10.1155/oti/6967523

**Published:** 2025-04-07

**Authors:** Elaine Judith Amaba, Catherine Joy Escuadra, Ji-Eun Choi, Sun-Joung Leigh An

**Affiliations:** ^1^Department of Rehabilitation Science, Graduate School of Inje University, Gimhae-si, Republic of Korea; ^2^Physical Therapy Department, College of Rehabilitation Sciences, University of Santo Tomas, Manila City, Philippines; ^3^HOPE Parent Training Center, Seoul, Republic of Korea; ^4^Department of Occupational Therapy, Inje University, Gimhae-si, Republic of Korea

**Keywords:** adaptive responses, graduate students, international students, occupational challenges, South Korea

## Abstract

**Background:** Humans, as occupational beings, are inherently driven to achieve mastery and adaptation. This drive is evident in international graduate students who face unique academic and sociocultural challenges when adapting to unfamiliar environments, such as South Korea. These challenges can hinder their occupational participation, yet there is a notable gap in the literature addressing the specific occupational issues faced by international students in this context.

**Aim:** This study is aimed at exploring the unique occupational challenges encountered by international graduate students in Korea and their general adaptive responses, analyzed through the lens of the occupational adaptation (OA) model. Additionally, this study proposed interventions for educational institutions and occupational therapy practitioners to support students in achieving OA.

**Method:** A scoping review was conducted following the PRISMA guidelines. Systematic searches across global (Web of Science, Scopus, and PubMed) and Korean (RISS and KISS) databases identified qualitative studies published in English or Korean over the past 10 years. ChatGPT-4 assisted with data extraction, which was reviewed for accuracy. Study characteristics were summarized using descriptive statistics, and thematic analysis identified key themes related to occupational challenges.

**Results:** The review identified 3 articles and 6 dissertations exploring the occupational challenges of 59 international graduate students in Korea. Most participants were master's students, primarily from China, Uzbekistan, and Laos. Two main themes emerged: (1) the hierarchical nature of Korean culture, which impeded academic and social engagement, and (2) the fast-paced lifestyle, which disrupted occupational balance. These challenges led to stress, isolation, and reduced participation, affecting role competence and quality of life. While students attempted adaptive strategies, they were often insufficient, highlighting the need for targeted interventions.

**Conclusions:** International graduate students in Korea faced significant pressure to achieve mastery in a demanding environment. Culturally sensitive interventions, combined with strategies like cognitive behavioral therapy (CBT) and lifestyle redesign, can improve stress management, self-advocacy, and occupational balance, leading to greater academic success.

## 1. Introduction

Humans, as occupational beings, possess a natural desire to engage in activities that enhance their physical and mental well-being [1]. For international students, defined as individuals pursuing education outside their homeland [2], this desire often drives them to seek educational opportunities abroad to advance their careers, foster personal growth, and access high-quality education, particularly in countries known for their academic reputation and economic opportunities [3, 4]. However, the journey of studying abroad is not without its challenges. International students often encounter barriers such as cultural differences, language limitations, and the high expectations of academic institutions, which complicate their adaptation process [3, 5].

Historically, international students have gravitated toward English-speaking countries like the United States, the United Kingdom, and Australia due to their globally recognized educational systems and the widespread use of English, a language familiar to many [6–8]. These countries have established themselves as popular destinations for higher education, providing a welcoming environment for students from diverse backgrounds. However, in recent years, South Korea (Korea) has emerged as a new educational hub, particularly for international graduate students [9, 10].

Korea's growing appeal can be attributed to its rapidly evolving educational system, advanced technological infrastructure, and rising reputation as a leader in innovation [11, 12]. Additionally, Korea's global cultural influence, particularly through the popularity of K-culture, and the government's efforts to internationalize higher education have contributed to its attractiveness as a study destination [13]. Yet, despite these positive attributes, international students, particularly those in graduate school, face greater challenges due to the demands of advanced studies and the need to adapt to a new environment, which can hinder their roles and overall participation [14, 15].

Occupational participation, a central concept in occupational therapy, refers to meaningful engagement in education, work, and social activities, all of which are essential for well-being and adaptation [16]. However, participation can be disrupted by various challenges, leading to difficulties in role fulfillment, a diminished sense of purpose, and reduced overall well-being [17–19]. In such circumstances, individuals must adapt to environmental demands to restore occupational participation and sustain engagement in their meaningful roles [20]. This need for adaptation is particularly evident among international students, who face multiple layers of transition—academic, social, and cultural—while striving to maintain their occupational engagement [21–23]. Despite these challenges, how international students navigate and sustain participation in these demanding contexts remains insufficiently explored, highlighting the need for further research into their adaptive responses.

Occupational therapists contribute to sustaining occupational participation by identifying barriers that disrupt engagement and guiding individuals in developing adaptive responses to enable continued participation [20, 24]. Barriers arise when individuals encounter change, transition, or disruption, creating a tension between their existing abilities and the new demands placed upon them, which necessitates adaptation [25–27]. The occupational adaptation (OA) model [28] explains this process further by defining this tension as a press for mastery, which emerges from the dynamic interaction between the person's internal desire for mastery and the environment's external demand for mastery. This press for mastery compels individuals to generate occupational responses or strategies aimed at overcoming challenges and meeting role expectations [29].

Occupational responses are generated by the person to meet environmental demands, but the ability to adapt varies as each person's adaptation gestalt—a combination of cognitive, psychosocial, and sensorimotor skills—differs in strength and development from person to person. These differences influence how well individuals adjust to occupational challenges, shaping their ability to refine and improve their previously generated occupational responses [29]. Whether an occupational response ultimately meets environmental demands depends on two key constructs: relative mastery and adaptive capacity. Relative mastery is achieved when the response is effective (achieves the intended goal), efficient (minimizes effort and stress), and personally satisfying (aligns with values and expectations) ([28], as cited in [30]). While measurable improvement in skills (adaptation gestalt) may not always occur, relative mastery can still be achieved if the individual perceives their response as effective, efficient, and satisfying, reflecting the complexity of adaptive responses, where satisfaction or acceptance of one's performance can be just as important as performance enhancement itself [31, 32].

However, mastery alone is not enough—long-term adaptation also requires adaptive capacity, or the ability to adjust and refine responses to meet changing demands. This capacity helps individuals develop adaptive responses that allow them to handle challenges and improve both academic performance and well-being [29]. When adaptive responses are overwhelmed by challenges that exceed capacity, dysadaptive responses may occur, making it harder to cope with academic and social pressures [33]. This highlights the need for support systems that encourage adaptive responses and prevent dysadaptive outcomes. Through this iterative process of adaptation, individuals work toward occupational adaptiveness, integrating refined responses into daily life, allowing them to manage challenges, maintain engagement in valued roles, and navigate changing demands across contexts [29].

For international students in Korea, this process of adaptation is particularly integral, as they must navigate both academic and cultural transitions simultaneously, yet research on their qualitative experiences remains limited. Existing studies have primarily relied on quantitative approaches, focusing on statistical trends such as stress levels, academic performance, and cultural adjustment difficulties [34]. While these studies provide valuable data, they fail to capture the lived experiences and adaptive processes that shape students' ability to adjust to new environments. Since international students have been found to possess greater awareness of the difficulties they encounter compared to other stakeholders [35], exploring their experiences in previous qualitative literature would provide valuable insights, particularly given the lack of studies examining how they actively engage in their occupations to adapt.

The OA model offers a relevant framework for understanding these adaptation processes, as it has been applied to role transitions in various populations. Studies have examined how immigrants adapt to new environments [36] and military personnel reintegrate into civilian life [37, 38]. However, most studies on occupational transitions among young adults focus on those with disabilities, such as individuals with disabilities and caregivers [39] or young adults with autism spectrum disorder [40]. When higher education students are included, research typically centers on college students, emphasizing stress and mental health rather than OA [41]. Studies on graduate students are rare, and the few that exist are outdated for research use [42].

Despite the OA model's applicability, its use in studying graduate students' adaptation in Korea has remained limited. These students have faced distinct academic, cultural, and professional challenges, requiring complex occupational adjustments that have been largely underexplored. These adjustments have shaped how students respond to adaptation demands, yet little research has examined their documented responses in the literature.

This scoping review identified these adaptation challenges and explored how international graduate students have navigated these challenges in existing studies. Understanding these challenges and responses provides insight into the adaptation process, informing future interventions and guidance on effective support strategies. This review was guided by the question: “What unique occupational challenges have international graduate students in Korea encountered, and what general adaptive responses have they employed as identified in the literature?”

## 2. Method

### 2.1. Study Design

This study utilized a scoping review methodology to systematically map the existing literature on the occupational challenges encountered by international graduate students in Korea. This approach was chosen for its ability to explore underresearched topics by synthesizing diverse published sources, including qualitative studies and dissertations, to provide a detailed and comprehensive overview while identifying specific gaps in the existing literature. The review was guided by established frameworks, particularly those proposed by Bennett et al. [43] and Lee and Kim [44], ensuring that the analysis was both rigorous and methodical, tailored specifically to meet the study's objectives. It also adhered to the PRISMA-ScR protocol [45], which guided the overall study design and ensured methodological rigor and transparency by structuring the review process in alignment with established scoping review standards (see Supporting Information (available here) for the PRISMA-ScR checklist).

### 2.2. Databases and Search Strategy

A comprehensive literature search was conducted across both global and Korean-specific academic databases to capture a wide range of relevant studies. Global databases such as Web of Science (WoS), Scopus, and PubMed were selected for their extensive collections of scholarly articles. Additionally, Korean-specific databases, including the Research Information Service System (RISS) and Korean Studies Information Service System (KISS), were utilized to ensure the inclusion of region-specific research. The search was conducted on June 6, 2024, and the results were finalized on June 20, 2024.

Keywords were derived from a prior systematic review by Cao and Meng [46] and adapted to capture terms related to international students, the Korean context, and challenges in adaptation and integration (see Table 1). Boolean operators and database-specific filters were applied to refine the search results ensuring relevance and specificity.

### 2.3. Selection Criteria

The inclusion criteria for the scoping review were defined to ensure both relevance and comprehensiveness. Articles and dissertations published in English or Korean within the past 10 years were included, specifically focusing on international graduate students (master's or doctoral levels) in Korea. Both peer-reviewed and nonpeer-reviewed sources were considered to capture a broad understanding of the occupational challenges faced by this population.

While quantitative and mixed-methods studies were available, the review focused exclusively on qualitative research to explore the depth and complexity of international graduate students' lived experiences. This focus allowed for the identification of nuanced, context-specific occupational challenges and adaptive strategies that qualitative research uniquely provides.

Most of the data were drawn from Korean databases, with a significant proportion of the included studies written in Korean. Researchers chose not to appraise the included studies, as the primary aim of this review was to explore themes emerging from existing research rather than assess the quality of individual studies. This decision reflects the need for greater visibility and cross-verification of findings in English and lays the groundwork for future systematic reviews and meta-analyses that can further strengthen the evidence base.

Exclusion criteria included studies focused on undergraduate students, research conducted outside the Korean context, sources unavailable in full text, and studies employing quantitative or mixed-methods designs, as these did not align with the study's objective of understanding specific experiential insights.

### 2.4. Data Gathering, Extraction, and Analysis

Two researchers conducted the data-gathering process, beginning with an initial search that identified 657 studies. Articles were first screened by title and abstract to assess their relevance, and full-text versions were retrieved for those meeting the preliminary criteria. Duplicate records were identified and removed during the data management phase. In cases where both articles and dissertations utilized the same datasets, the article version was selected for inclusion due to its typically more rigorous peer review process. Discrepancies during screening were resolved through consensus discussions. The study selection process followed the PRISMA guidelines [47] to ensure transparency and replicability, with the flow diagram visually depicting the inclusion and exclusion process.

Data extraction was conducted independently by the same researchers, focusing on key details such as study objectives, demographics, methods, and findings. To ensure alignment with the research questions and relevance to occupational therapy, two additional researchers with occupational therapy backgrounds counter-checked the extracted data. ChatGPT-4 was employed to assist in organizing the extracted data, following the approach described by Alshami et al. [48].

**Figure 1:**
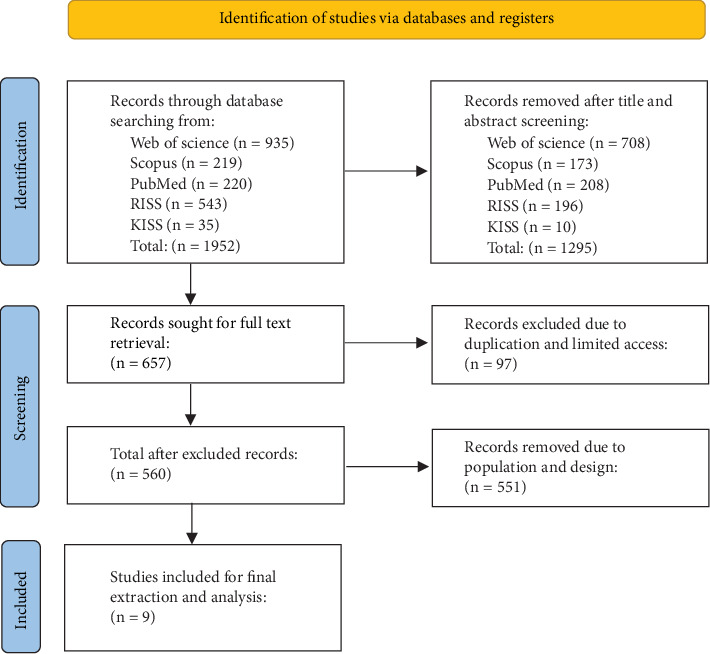
PRISMA diagram.

Subsequently, data analysis was carried out in two stages. First, descriptive statistics were performed using MS Excel and RStudio to summarize study characteristics and establish a foundation for qualitative synthesis. Next, thematic analysis was conducted using Delve software [49], involving iterative coding and refinement into broader themes. To ensure cultural relevance and enhance methodological triangulation, these themes were then reviewed by the same two additional researchers, both of whom are native Korean occupational therapists. Weekly consensus meetings were held to resolve coding discrepancies and validate the themes (see Figure 1 for the PRISMA diagram flow chart).

### 2.5. Coding and Theme Development

The coding process involved systematically analyzing and categorizing excerpts from the transcripts to capture recurring patterns. For example, the quote, “With elders, we need to use the honorific form to talk to them. We cannot use ‘요' instead of ‘습니다,'” was initially coded as “language rules” and later grouped into the subtheme: “honorifics, titles, and social norms,” which contributed to the broader theme of “the hierarchical culture.”

Given the limited number of included studies, some codes did not have enough data from varying participants to form independent themes. Therefore, the research team used premade categories such as “occupational challenges” and “adaptive responses” to initially filter and organize the data in line with the OA model. These provisional themes were later revised to better reflect the core challenges and adaptive responses identified in the data.

Subthemes were created only when supported by data from at least four different studies, ensuring reliability. When adaptive responses were not explicitly linked to specific challenges, they were grouped under the general theme “adaptive responses” due to the nature of the transcripts, which did not always specify which response was connected to which challenge. This approach helped maintain clarity and consistency while organizing the data within the overarching framework.

## 3. Results

The scoping review identified and analyzed a total of 3 articles and 6 dissertations employing qualitative methodologies to explore the occupational challenges faced by international graduate students in Korea. The studies primarily utilized purposive sampling methods, with 46 participants (77.97%) recruited through this approach. Data were collected from 59 participants, comprising 47 master's students (79.66%) and 12 PhD students (20.34%). The gender distribution was 16 males (27.12%) and 43 females (72.88%), with an average age of 26.56 ± 1.45 years. Percentages are provided in parentheses for descriptive purposes only, reflecting participant demographics without quantifying individual experiences.

### 3.1. Participant Demographics

Table 2 presents the distribution of the participants' nationalities specifically focused only on the qualitative studies reviewed. The majority of participants originated from China (*n* = 19), Uzbekistan (*n* = 13), and Laos (*n* = 11), highlighting a significant representation from Asian countries, particularly from regions with close geographical and cultural ties to Korea.

### 3.2. Study Designs

In terms of study design, a significant proportion of the included studies utilized case studies and narrative inquiry methodologies, each accounting for 3 (33.33%) of the total. These approaches were followed by studies categorized as qualitative without a specified design, comprising 2 (22.22%), and phenomenological studies, which made up 1 (11.11%). This distribution suggests a need for greater methodological variation and the incorporation of additional strategies to more comprehensively capture the diverse experiences of international graduate students in Korea. A summary of the main features and findings of the included studies is provided in the Appendix (see Table A1).

### 3.3. Thematic Analysis Results

This thematic analysis examined the unique OA experiences of international graduate students in Korea, as reported in the nine reviewed studies. The focus was on identifying challenges specific to the Korean context, deliberately excluding issues commonly faced by international students globally. Two main themes emerged:

1. The hierarchical culture
2. Navigating Korea's fast-paced culture

These themes were selected based on their frequent occurrence across the studies, their cultural relevance, and their observable impact on students' ability to engage in academic, social, and daily occupations, as evidenced by challenges like suppressed abilities and reduced meaningful participation.

#### 3.3.1. Theme 1: The Hierarchical Culture

The first theme, the hierarchical culture, highlights how Korea's strong emphasis on hierarchy creates substantial occupational challenges for international graduate students. This cultural norm impacts students' ability to adapt and effectively navigate their roles as graduate students and members of society.

##### 3.3.1.1. Hierarchical Authority in Academic Relationships

One of the primary challenges for international graduate students in Korea was the expectation to respect and defer to authority figures, particularly professors. This dynamic often discouraged students from actively engaging in academic discussions, as they tended to “unconditionally follow and agree with the professor” [53]. This pattern of behavior was also observed during group activities, where students refrained from expressing differing opinions and instead aligned with the perceived authority or majority [55, 57]. A Saudi Arabian student further emphasized the pressure of this hierarchy, explaining that they felt obligated to “make older people feel valued” [58], which further complicated communication and mentorship with professors.

As a result, some students struggled to connect with their advisors. One student shared, “I don't know how to communicate with them and have to be very cautious. They are very authoritative, which is tough” [53]. This lack of communication and support often left students feeling isolated, with some perceiving their professors as “detached,” which resulted in less guidance and support [53].

Cultural differences in mentorship styles also contributed to this challenge. For example, students from China noted that their home country professors were more approachable and provided detailed research guidance, whereas Korean professors emphasized student autonomy [53]. Similarly, students from Uzbekistan observed that professors in their home country “checked on students more often” [58]. These differences in academic expectations contributed to stress, anxiety, and isolation among the international students, as the hierarchical environment suppressed critical thinking and self-expression [53, 58].

To adapt, many students adopted a more “self-reliant” and “independent” approach, accepting their professors' expectations and adjusting to the demands of Korean academic culture [51, 53, 55, 57]. Some embraced this as part of adapting to Korean society, understanding that respect for authority and elders was deeply rooted in Confucian values [58].

##### 3.3.1.2. Honorifics, Titles, and Social Norms

Language barriers, particularly the use of correct honorifics, added another layer of difficulty for international students in navigating Korea's hierarchical culture. Misusing honorifics led to misunderstandings and negative impressions, as one student explained: “miscommunication can happen, and it can give a negative impression, such as being rude or disrespectful” [57]. This challenge affected students' ability to form meaningful social and academic connections.

For instance, a student from Uzbekistan found it challenging to manage the varying levels of formality, stating, “When I talked to my friend and seonsaengnim (teacher) at the same time, I had to know when to use banmal and when to use jondaenmal. That was hard” [58]. Similarly, a Lao student noted stricter use of honorifics with elders in Korea, compared to Lao culture, where respect is shown more through “posture” than language [52].

The complexity extended beyond language. A Pakistani student expressed confusion over following orders simply based on someone's age or rank [58]. Additionally, the directness required in the Korean language structures added to the difficulty. A Saudi Arabian student found the grammar used for requests—such as “*juseyo*” (please give me)—to be culturally “weird” compared to more indirect forms of asking in their culture [58].

Faced with these challenges, students had no choice but to adapt to Korean social norms. An Uzbekistani student regarded these expectations as a reflection of basic manners, similar to those in their home country, while an Italian student acknowledged the need to “humble oneself” as an essential part of demonstrating politeness [58].

Beyond language, the rigid use of titles, such as addressing each other as “teacher,” exacerbated feelings of isolation. One student described the academic environment as “individualistic,” stating, “at graduate school, everyone calls each other ‘teacher.' I don't feel like we are sharing anything together” [50]. This formal use of titles and grammar reinforced social hierarchy, making it harder for students to build supportive networks, which were crucial for well-being.

In response, many students sought social integration by forming friendships within their own national communities, staying connected with foreign groups, reaching out to their family members, or attending church to regain a sense of belonging [50, 52, 55].

#### 3.3.2. Theme 2: Navigating Korea's Fast-Paced Culture

The second theme, navigating Korea's fast-paced culture, explored the occupational challenges and advantages arising from the country's emphasis on speed and efficiency, embodied in the concept of *ppalli-ppalli* (hurry–hurry).

##### 3.3.2.1. Fast-Paced Academic Environment

Many students found it difficult to cope with the rigorous academic demands and long work hours in their graduate laboratories, which often disrupted their ability to manage other aspects of life and maintain occupational balance. Some Chinese students expressed that they “can't keep up with the research workload” [53], with language barriers exacerbating the issue. As one student explained, “I need to read many papers, but the difficulty is too high” due to their “Korean language skills not being good enough to fully understand the papers” [54]. This often led to the practice of starting one paper before finishing the previous one, making it harder to keep pace with professors' expectations [54]. These experiences illustrate how the fast-paced academic environment can lead to significant occupational stress.

The demanding workload in Korea significantly impacted students' sleep, well-being, and overall productivity. A Lao student shared that their lab professor advised them to sleep “less than four hours,” reinforcing the stereotype that “Korean people sleep only four hours,” which added immense pressure to work harder. This pressure was further compounded when a Korean peer informed them that they were expected to work in the lab from 9:00 a.m. to 10:00 p.m., leaving the students shocked and questioning how they could sustain such long hours [52]. Similarly, other students criticized these practices, pointing out that the overwhelming workload and long hours not only affected their health but also created a work culture that was “not productive,” often resulting in diminished efficiency and productivity [58].

Despite these challenges, some students saw the fast-paced environment as an opportunity for personal growth. An Uzbekistani student reflected on how the demanding academic setting helped them develop discipline and work ethic, stating, “The more demanding the professor, the smarter I become, so I'll work hard” [56]. Another student highlighted the importance of meeting deadlines, remarking, “After coming to Korea, I made an effort to keep my commitments with the professor. I think it would be great for Uzbek people to learn about keeping promises from Koreans” [56]. These experiences suggest that while the fast pace initially presents difficulties, it can foster resilience, improved time management, and a stronger sense of responsibility.

## 4. Discussion

**Figure 2:**
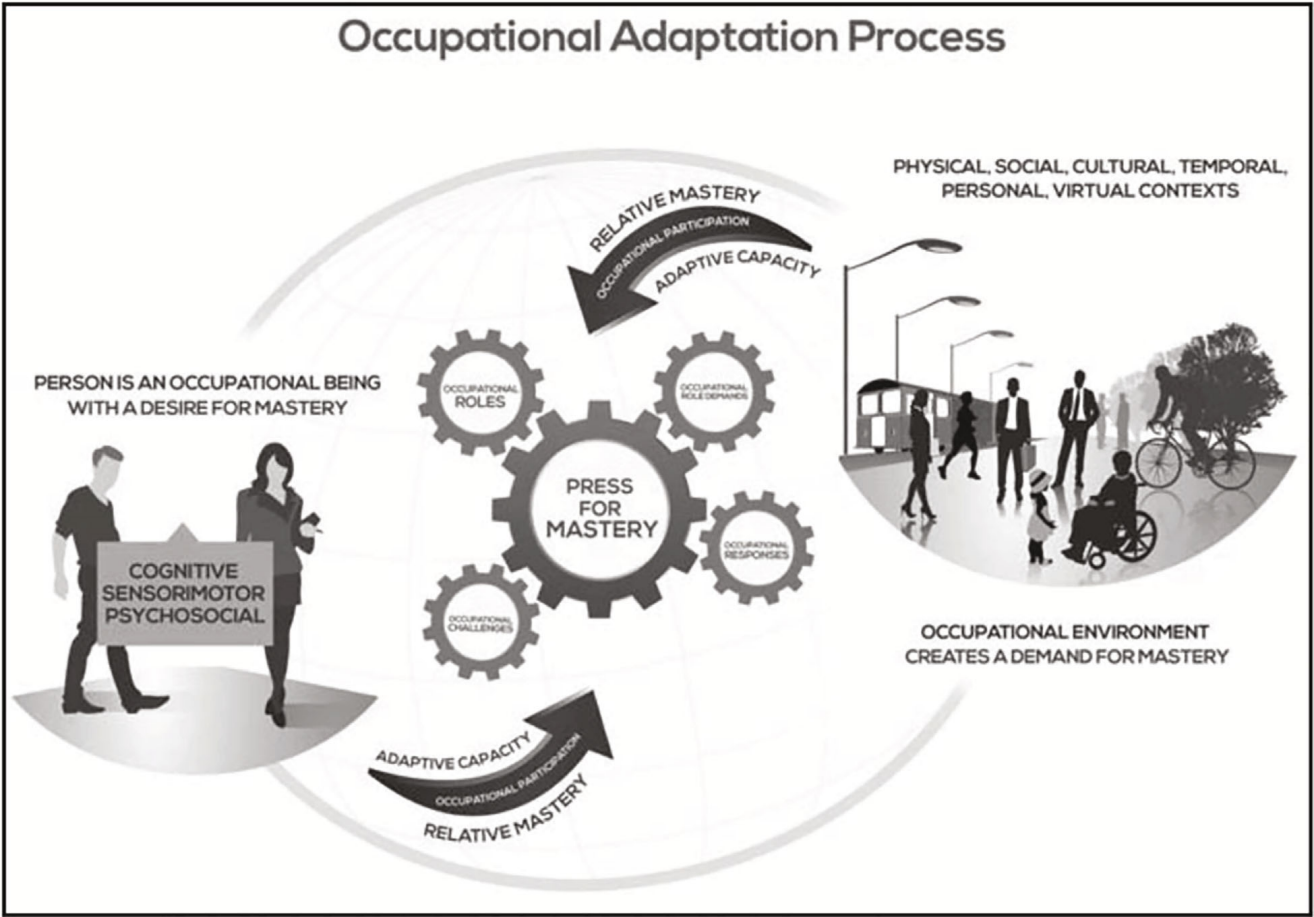
A reconceptualization of Schkade and Schultz's OA process [20].

This scoping review examined the occupational challenges and adaptive responses of international graduate students in Korea, focusing on how their interactions with the academic environment shaped their adaptation process. The thematic analysis identified two main challenges: the hierarchical culture and navigating Korea's fast-paced culture. These findings provide a foundation for discussing the interplay between students' desire for mastery and the environmental demands, which create the press for mastery through the interaction or tension between their occupational roles, role demands, occupational challenges, and occupational responses. Their adaptive responses, framed through the OA model as an internal normative process, will also be explored. Furthermore, the discussion will highlight how these factors collectively either support or hinder students' adaptation efforts (refer to Figure 2 for a visual representation of the adaptation process, which provides a guide for understanding OA model terms and the subsequent discussion).

### 4.1. The Person: Desire for Mastery and Adaptation Gestalts

International graduate students demonstrated a strong desire for mastery, motivated by their ambition to meet rigorous academic and cultural expectations. However, this drive often resulted in a mismatch between their adaptation gestalts—cognitive, psychosocial, and sensorimotor skills—and the hierarchical and fast-paced demands for mastery of Korea's academic culture in its occupational environment. This mismatch was particularly challenging for students from non-Confucianist cultures, who struggled with deeply ingrained cultural norms emphasizing deference to authority and hierarchy, a central tenet of Confucian values [59]. These findings are consistent with previous research linking cultural unfamiliarity to heightened stress among international students [60].

To navigate these demands, students employed adaptive strategies, such as reframing hierarchical norms and academic pressures as opportunities for growth. This approach, supported by cognitive restructuring and stress management literature techniques [61, 62], enabled students to reduce psychological distress and maintain engagement despite the pressures of their environment. Such findings align with the OA model's focus on adaptive capacity, where individuals modify thoughts, behaviors, or skills to meet environmental demands [29], emphasizing the need to support students' adaptation in culturally demanding contexts.

### 4.2. The Occupational Environment: Cultural Demands and Adaptation

The concept of the occupational environment in the OA model parallels the environment described in OTPF-3, encompassing cultural, social, physical, temporal, virtual, and personal contexts [20, 27, 63]. However, while global studies often focus on physical (food change, dress codes, university facilities, climate, and safety) and temporal changes experienced by international students [64–67], this review highlighted the unique impact of Korea's cultural and social contexts. Hierarchical norms, rigid expectations, and the fast-paced academic culture created significant challenges, leading to reduced engagement in academic activities, social isolation, and a lack of guidance [50, 52, 53, 58]. These findings reflect research that identifies cultural barriers as intensifying adaptation difficulties by limiting social connection and role clarity [68].

Building relationships within national and international student communities proved effective in alleviating these challenges. These connections provided emotional support, shared experiences, and practical advice, which reduced cultural stress and fostered mutual understanding [69, 70]. Informal peer networks also offered guidance in navigating hierarchical norms and fast-paced expectations, allowing students to adapt socially and academically. However, further exploration is needed to determine how institutional support, such as culturally tailored mentoring programs, could complement peer-based strategies to enhance adaptation outcomes [69, 71, 72].

### 4.3. Press for Mastery: Occupational Role, Role Demands, Challenges, and Responses

The press for mastery, as conceptualized in the OA model, arose from the tension between students' occupational roles and the demands of Korea's academic culture. These roles required students to meet high academic expectations while adhering to hierarchical norms and managing the fast-paced academic environment. This tension was amplified when students' skills did not align with these demands, resulting in barriers such as language difficulties, rigid academic structures, and insufficient institutional support. These challenges often led to dysadaptive responses, including withdrawal, avoidance, and reduced participation in academic and social activities [50, 52, 53, 58], consistent with international student's experiences and findings globally [73, 74]. The adaptation process was not uniform but varied from person to person, reflecting the trial-and-error nature of adaptation [20] as students continuously adjusted their responses based on how well their skills aligned with environmental demands. Prolonged periods of dysadaptation further underscored the intensity of these challenges, highlighting the critical need for targeted support.

Despite these barriers, enablers such as strong peer networks, informal mentoring relationships, and access to academic resources played a critical role in supporting students' adaptation [50, 52, 53, 58]. These strategies provided emotional reassurance, practical advice, and shared experiences, as seen in the research by Caligiuri et al. [75]. While these supports helped students navigate their roles and reduce cultural stress, further research is required to explore how adaptive capacity and relative mastery—key constructs in the OA model—shape international students' perceptions of success and satisfaction in their adaptation. Occupational therapists can facilitate these processes through culturally sensitive interventions, including stress management workshops and peer mentoring, aligning with the OA model's goal of supporting state of adaptiveness [20].

### 4.4. Facilitating OA

Occupational therapists can facilitate international graduate students' adaptation by addressing the specific barriers and enablers identified in this study. Using the OA model as a guiding framework, occupational therapists can empower students to strengthen their adaptive capacity, equipping them with tools and strategies to navigate Korea's hierarchical and fast-paced academic environment. Rather than prioritizing skill improvement and development alone, occupational therapists can foster a client-centered, role-focused approach that emphasizes awareness of progress and satisfaction [20], encouraging students to view adaptation as a dynamic process rather than a measure of perfection.

To support this process, interventions such as cognitive behavioral therapy (CBT)–based stress management training [61, 62, 76–78] can help students reframe academic pressures and cultural challenges as opportunities for growth, aligning with the OA model's emphasis on adaptive responses. A key feature of CBT, cognitive reframing or restructuring, helps individuals reinterpret challenges, such as academic workloads or language barriers, in ways that foster resilience and adaptation [76, 78]. Additionally, lifestyle redesign [79, 80], focused on balancing academic demands, self-care, and stress management, can further enhance their occupational balance. Together, these approaches not only address individual needs but also promote long-term adaptation and well-being.

At the institutional level, culturally sensitive programs can create a supportive environment for international students [81]. Tailored Korean language classes, designed for hierarchical and academic contexts, could reduce cognitive strain by enabling students to navigate formal communication more effectively. Additionally, peer mentoring programs that pair culturally competent mentors with new students can provide emotional reassurance, practical advice, and guidance [82–84] on adapting to Korea's academic culture. Cross-cultural communication workshops for all staff members [84, 85] can also foster inclusivity and mutual understanding, ensuring that institutional practices align with the diverse needs of international students.

By implementing these interventions, occupational therapists, educators, counselors, and administrators can help students achieve relative mastery—where occupational responses are not only effective and efficient but also personally satisfying for the individual [20]. These efforts align with the OA model's emphasis on the engagement in meaningful participation and empower students to thrive academically and socially in Korea's unique cultural context.

## 5. Limitations

This review has several limitations that highlight important research gaps. The small number of qualitative studies and the absence of formal quality assessments may have overlooked methodological weaknesses, reducing the reliability of the findings. Additionally, most studies relied on purposive sampling and predominantly included Asian participants, limiting the diversity and generalizability of the results to other cultural contexts. While this review identified significant challenges, detailed insights into adaptive strategies were lacking, as these were not well-documented in the included papers. Furthermore, the absence of longitudinal studies restricts understanding of how adaptation evolves over time, leaving a gap in knowledge about long-term adaptive processes. Finally, the occupational challenges identified were largely academic, with minimal exploration of challenges in nonacademic contexts.

## 6. Recommendations

Future research should explore how international students' adaptive responses are shaped, focusing on whether these are adaptive or dysadaptive, along with the process of adaptive capacity and relative mastery in specific detail. Longitudinal and mixed-methods studies are recommended to capture the dynamic nature of adaptation and provide deeper insights. Beyond academics, research should investigate other occupational domains, identify key barriers and enablers, and evaluate interventions like lifestyle redesign and CBT to enhance adaptability and well-being. Expanding studies to diverse countries, addressing the distinct needs of doctoral and master's students, and increasing English publications would improve generalizability and highlight the critical role of occupational therapists in fostering adaptation.

## 7. Conclusion

This scoping review identified the occupational challenges faced by international graduate students in Korea, highlighting their general adaptive responses through the framework of the OA model as an internal normative process. The findings indicated that Korea's hierarchical culture and fast-paced academic environment imposed significant adaptive pressures, often resulting in dysadaptive responses such as withdrawal, isolation, and reduced academic engagement. While the OA model provided a valuable lens for analyzing these challenges, its application in the context of international higher education remains limited, warranting further research to explore its broader utility and refine its conceptual framework. The review also emphasized the potential role of occupational therapy practitioners and educational stakeholders in delivering culturally sensitive interventions to enhance students' adaptiveness. Future studies should expand the application of the OA model to better understand its relevance and impact in addressing the unique demands of international students in highly structured academic settings.

## Figures and Tables

**Table 1:** Summary of keywords utilized for searching.

**English keywords**	**Korean keywords**
• KEY 1[(international student) OR (overseas student) OR (foreign student)] • KEY 2 [(South Korea) OR (Republic of Korea) OR (Korea)] • Key 3 [(universit⁣^∗^) OR (tertiary institution) OR (higher education)] • KEY4 [(transition OR adjustment OR adaptation OR integration OR adjustment OR assimilation OR coping OR fit OR satisfaction OR depression OR stress OR anxiety OR loneliness OR wellbeing OR belonging OR connectedness OR academic⁣^∗^ OR learning⁣^∗^)]	• KEY1:유학생 • KEY2: 한국 • KEY3: 대학교 • KEY 4: 생활 • KEY 5: 적응

**Table 2:** Country of origin of the participants of the collected qualitative studies.

**Participants (country)**	**Total**
China	19
Uzbekistan	13
Laos	11
Pakistan	3
Kyrgyzstan, Russia, Vietnam	2
Azerbaijan, Egypt, Philippines, United States, Nigeria, Saudi Arabia, and Italy	1

**Table 3:** Summary of the main features and findings of the included studies.

**Author and year**	**Aim**	**Document type/research design/sampling/language**	**Sample**	**Results**
Kim [50]	This paper inquires into the experience of two Chinese international graduate students who speak fluent Korean in the aspects of social connectedness and social support.	Article Narrative Inquiry Purposive sampling Language: Korean	Sample size: 2 Gender: 2 females Age: 25–27 years old (mean: 28.5) Nationality (per country): China—1 Vietnam—1 Year level: 2 masters	Participant features were identified as follows: First, their social connectedness in the university community was not secured, and social support, except for informational support, was also not solid. Second, they perceived their conformity group as social members outside the university community, where social connectedness and social support were provided and vice versa. Third, their intimate experience of belonging as a member of the social connectedness and the mutual support provided in the social connectedness rewarded them with stability that generated a positive influence on their international student experience. Findings from this analysis suggest that there is a need for requisite change in the perception and support for international students in the Korean university community.

Sevara [51]	The purpose of this study is to investigate the academic experiences of graduate students from Uzbekistan currently residing in Korea and to highlight their significance.	Dissertation Case Study Snowball sampling Language: English	Sample size: 9 Gender: 4 males; 5 females Age: 24–28 years old (mean: 26) Nationality (per country): Uzbekistan Year level: 5 masters; 4 doctorate ⁣^∗^Some are scholars	First, personal training is necessary to assist students, professors, seniors, and juniors in the lab in building strong personal relationships with others. Second, essential cultural orientation classes are required to help students learn specific cultural norms in Korea, such as expected roles in the classroom, social behavior, and other cultural characteristics unique to Korean society. Third, Korean language courses for graduate students are crucial for a successful graduate study experience. Finally, this study suggests that similar research should be conducted not only for graduate students from Uzbekistan and other foreign students but also for Koreans. As Korea becomes a more multicultural society, it is important for Koreans and people from other cultures to coexist harmoniously, with both sides needing to develop cultural understanding. This study is expected to serve as a tool to further strengthen the relationship between Uzbeks and Koreans.

Sonbouttasene [52]	To explore on reasons Lao international students study in Korea, four adjustment difficulties, and degree of satisfaction based on their experience in South Korea.	Dissertation Case Study Snowball sampling Language: English	Sample size: 11 Gender: 6 males; 5 females Age: 23–40 years old (mean 27.64) Nationality: Laos Year level: 11 masters ⁣^∗^All under scholarships	The most optimistic findings have emerged in general living adjustment, which relates to scholarship benefits, financial management, savings, and part-time jobs. Yet, the major findings show that academic adjustment is the most challenging, comprising academic performance, language issues, and a new system of education. As a result, personal–psychological adjustment has shown that Lao postgraduates experience stress from academic adjustment, and some of them feel lonely because of their introverted character. In social–cultural adjustment, most Lao postgraduates reported that they have never experienced racial discrimination because of their social science major, type of scholarship, and self-study. Contrary to the majority, two students, science majors under professor scholarships, have revealed that they confronted racial discrimination among professors and Korean students. To overcome academic difficulties, respondents mention personal effective strategies such as being well-organized, well-focused, and time-managed, as well as practicing self-discipline and self-study in their studies. Various activities in exercise and religion have been revealed as major contributors to physical and mental health. Furthermore, social supports directly/indirectly influenced the improvement of Lao students' academic skills. Although the study participants dealt with many difficulties, the majority reflect satisfaction with studying in South Korea rather than dissatisfaction. Ultimately, even if most postgraduates decide to return to their home country or work in a host country, they are considered cultural diplomacy or brain drain.

Lim [53]	The current study is aimed at exploring the major challenges non-STEM Chinese doctoral students encounter during their academic socialization process at a research-focused university in Korea.	Article Case Study Purposive sampling Language: Korean	Sample size: 10 Gender: 2 males; 8 females Age: 20s (unspecified) Nationality (per country): China Year level: 10 masters	First, Chinese doctoral students were “marginalized” in their academic community due to weak academic and emotional interactions with their advisor (hierarchical relationship and neglected supervision style) and peer students (unequal power relationship between local vs. international students). Also, they were excluded from participation opportunities in various tasks and roles (e.g., research projects), which intensifies their marginalized status in the academic community. Major strategies to resolve these difficulties were, first, to utilize various internal and external resources needed to conduct research “independently”; second, to utilize the network of other foreign student peer groups to strengthen both emotional and academic support; and third, to utilize their network with the Chinese academic community from their past educational experience, as well as with overseas researchers through participating in international academic conferences. Based on these findings, the study provides implications for the need for effective academic socialization of international PH.D. students and related improvements in the rapidly internationalizing Korean doctoral education system.

Li [54]	To deeply understand the therapeutic song-writing experiences of Chinese international students with enculturation stress and study stress.	Dissertation Phenomenological Purposive sampling Language: Korean	Sample size: 5 Gender: 5 females Age: 28–34 years old Nationality (per country): China Year level: 5 doctorate	The five components were “difficulty in daily life,” “difficulty in study,” “positive experience in activities,” “difficulty in activities,” and “positive change through activities.” As a result of the study, Chinese international students were able to express their emotions through therapeutic songwriting activities. The participants faced challenges in the creative process and overcame difficulties and conflicts to the end. This process gave participants a sense of accomplishment. They also gained sympathy and support by creating and sharing with their colleagues. The positive experience of participating in these therapeutic songwriting activities provided them with a meaningful way to adapt to studying in Korea.

Konul [55]	To explore the adaptation process and factors influencing the adaptation of international students to Korean university life, focusing on international students studying at Yonsei University	Dissertation Narrative Inquiry Purposive sampling Language: Korean	Sample size: 10 Gender: 2 males; 8 females Age: not specified Nationality (per country): Russia—1 Azerbaijan—1 Egypt—1 Philippines—1 Kyrgyzstan—2 Pakistan—2 United States—1 Nigeria—1 Year level: 10 masters	Firstly, academic difficulties and academic stress were found to play important roles in the adaptation process of international students. Secondly, in relation to cultural differences, international students demonstrated a sense of distance between Korean culture and their own culture. Thirdly, when faced with difficulties, international students primarily sought help from family and friends, and they also received assistance from friends from the same country. Fifthly, it was confirmed that international students utilized university counseling services. The participants viewed the counseling services as part of their academic life and utilized them to cope with problem-solving and academic challenges. Lastly, the analysis of the adaptation level of international students at Yonsei University revealed that the majority aligned with Category 4b according to G. Hofstede's U-shaped cultural assimilation theory. This indicates that international students generally adapt well to Korean university life.

Park [56]	To explore and analyze the preparation processes and initial adaptation experiences of three Uzbek female master's students studying at universities in Korea.	Article Qualitative (Unspecified) Purposive sampling Language: Korean	Sample size: 3 Gender: 3 females Age: 21–27 years old (mean: 23.67) Nationality (per country): Uzbekistan Year level: masters	First, visa issuance is difficult and complicated, and debts owed must be paid in the future. Second, they solve meals with difficulties in finding accommodation and delivering food. Third, academic difficulties must be overcome due to unfamiliar Korean skills. Fourth, although they are getting used to Korean culture, Koreans' prejudice is a burden. Even though this study has a limitation that the participant in this study is just three international students, the study results can be used for research materials for improvement of policy or system for international students. Further research on university life conducted on a large number of graduate students is expected to be performed so that international students can lay the foundation for growth through education in Korea.

Lee [57]	First, the research questions for the purpose of this study are as follows: First, what is the motivation of foreign students to study abroad in Korea? Second, what are the difficulties in adapting foreign students to Korean society? Third, what is the process of overcoming these difficulties? Fourth, how are international students adapting to Korean society through daily learning? Fifth, what kind of learning has been done in Korean society? Sixth, how has learning helped solve these problems? Seventh, a research question was set up by discussing future hopes or dreams.	Dissertation Narrative Inquiry Purposive sampling Language: Korean	Sample size: 3 Gender: 3 females Age: 26–29 years old (mean 27 years old) Nationality (per country): China Year level: 3 doctorate	First, it was found that academic difficulties and academic stress play an important role in the adaptation process of foreign students. These difficulties negatively affect the welfare and social life of foreign students, and it was found that they experienced a longing for their hometown and homesickness due to academic stress. Second, regarding cultural differences, it was shown that foreign students felt something between Korean culture and their own culture. These cultural differences act as a challenge faced by foreign students in interpersonal relationships and induce negative emotions such as loneliness, longing for their hometown, and isolation. Therefore, it was found that appropriate support was needed to detect and overcome cultural distance. Third, in this study, it was found that foreign students first asked for help from their families and friends and received support from friends from the same country. In addition, it was found that they tried to obtain intimacy and emotional help in relationships with compatriots. This shows that foreign students tend to receive support from family and friends due to their cultural attitude when solving problems. Fourth, in this study, it was also confirmed that foreign students are using graduate counseling services. Participants considered university counseling services as a part of their academic life and were using them to solve problems and cope with academic challenges. As for the attitude toward these counseling services, it was found that access through various channels had a positive effect, and satisfaction was generally high. Research participants value support and help from family, friends, and colleagues, and it can be seen that factors such as language barriers, academic difficulties, and cultural differences have a major influence on the adaptation process. In consideration of this, universities and educational institutions need to take measures such as providing appropriate support and programs for foreign students. In addition, it will be possible to support foreign students' academic and cultural adaptation through efforts such as providing counseling services and strengthening cross-cultural understanding.

Daria [58]	The study is aimed at researching why international students come to Korea, their life here, how well their adaptation process to the language and culture goes, what aspects of language are the most difficult, and how they deal with it.	Dissertation Qualitative (unspecified) Purposive sampling Language: English	Sample size: 6 Gender: 2 males; 4 females Age: 23–30 years old (mean 26) Nationality (per country): Russia—1 Uzbekistan—1 Saudi Arabia—1 Italy—1 Pakistan—1 Vietnam—1 Year level: 6 masters Length of stay: 6–24 (mean 15 months) Some are scholars	The result shows that the majority of interviewees consider the quality of South Korean education to be very high. At the same time, most interviewees feel pressure to get along well in Korean society. The results of this study can be used in making policies and guidelines for international students living in South Korea. It would help South Korean universities to understand and consider the needs of international students from different countries. It will then lead to the creation of a better academic environment for higher education in South Korea.

*Note:* The asterisk (⁣^∗^) is used to indicate that the participants in this study received some form of scholarship—whether full, partial, or another type of financial support related to studying abroad. This asterisk notation should also be seen in the works of Sevara, Sonbouttasene, and Daria, where similar distinctions were made.

## Data Availability

The data that support the findings of this study are available from the corresponding author upon reasonable request.

## Supporting Information

Supporting Information Additional supporting information can be found online in the Supporting Information section. This study followed the PRISMA-ScR (Preferred Reporting Items for Systematic Reviews and Meta-Analyses extension for Scoping Reviews) checklist to ensure methodological transparency and rigor [44]. This checklist serves as a standardized framework, ensuring a systematic and comprehensive approach to reporting scoping reviews.
